# Early application of extracorporeal membrane oxygenation in influenza B virus-related fulminant pneumonia complicated with methicillin-sensitive *Staphylococcus aureus* infection: a case report

**DOI:** 10.3389/fmed.2025.1549856

**Published:** 2025-04-16

**Authors:** Hong Ding, Jia-ding Xia, Xin Zheng, Zi-yan Wang, Kun Zhang

**Affiliations:** Department of Intensive Care, Affiliated Hospital of Chengde Medical University, Chengde, China

**Keywords:** influenza B virus, methicillin-sensitive *Staphylococcus aureus*, lethal pneumonia, septicemia, extracorporeal membrane oxygenation

## Abstract

Co-infection of the influenza B virus with other bacterial pathogens is a significant contributor to the high pathogenicity and mortality associated with influenza B. The most common bacterial co-infections are caused by *Staphylococcus aureus* and *Streptococcus* species. In this case report, we describe the clinical symptoms and treatment of a 69-year-old woman who developed fulminant pneumonia secondary to *S. aureus* infection following initial influenza B virus infection. This case emphasizes the importance of early recognition and the use of extracorporeal membrane oxygenation in treating fatal pneumonia caused by co-infection with methicillin-sensitive *S. aureus* and influenza B virus. We conclude that this case provides valuable insights into the severe complications of influenza co-infections and underscores the role of extracorporeal membrane oxygenation in the management of fulminant pneumonia.

## 1 Introduction

Influenza is an acute respiratory infection caused by influenza viruses, with influenza B virus (IBV) being a major pathogen. IBV infections account for approximately 23% of all influenza cases. Post-influenza bacterial infections are the leading cause of mortality in influenza virus infections (1). Morens et al. demonstrated that, during the 1918 Spanish influenza pandemic, over 90% of influenza patients had bacterial co-infections, primarily with *Staphylococcus aureus*, *Streptococcus pneumoniae*, and other pathogens (2). However, the clinical characteristics of IBV co-infected with *S. aureus* remain underexplored. This report presents the clinical features and treatment of an adult patient who developed severe IBV-related pneumonia complicated by methicillin-sensitive *S. aureus* (MSSA) infection. The patient was admitted to the Affiliated Hospital of Chengde Medical College in January 2024. This case aims to enhance awareness and improve treatment success rates for similar severe infections.

## 2 Case presentation

A 69-year-old woman with a history of coronary artery disease presented with a 5-day history of cough, sputum production, and a 1-day history of fever and dyspnea. Her symptoms began 5 days prior with a cough producing white sputum and myalgia, without any obvious cause. 1 day before hospitalization, her condition worsened, with increased coughing, sputum production, and fever (38.2°C), along with respiratory distress and oliguria (24 h < 600 mL). She was initially admitted to Fengning County Hospital but was transferred to the emergency room of the Affiliated Hospital of Chengde Medical College due to persistent respiratory difficulties. Upon admission to the ICU, the patient’s dyspnea rapidly worsened, necessitating endotracheal intubation. Her physical examination upon ICU admission revealed the following: Body Mass index (BMI) of 18.7, temperature of 35.2°C, heart rate of 128 bpm, blood pressure of 95/60 mmHg, and oxygen saturation of 90% on 100% oxygen (FiO2) and positive end-expiratory pressure (PEEP) of 12 cmH_2_O. She was analgesia and sedation, with bilateral pupils measuring 3.0 mm and delayed light reflex. Auscultation revealed weak respiratory sounds in both lungs, with the presence of wet rales. Peripheral extremities were cool, and cyanosis was observed in the nail beds of the fingers. Her lower extremities showed signs of skin mottling.

**Figure 1:**
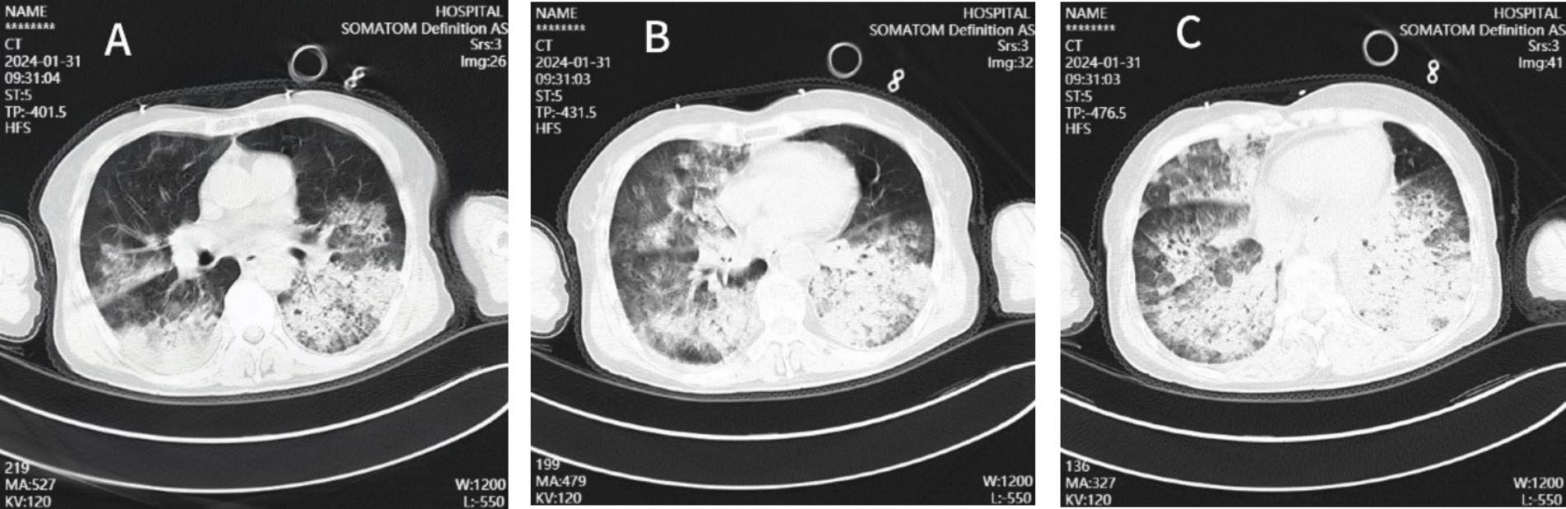
Chest CT images of the patient. The CT chest images **(A–C)** showed patchy shadows and ground-glass shadows in the lungs.

Laboratory results included blood gas analysis showing a pH of 7.42, pCO_2_ of 50 mmHg, pO_2_ of 50 mmHg, and base excess of −11.9 mmol/L. Biochemistry tests revealed AST: 4,122 IU/L, AST: 1,739 IU/L, total bilirubin: 13.11umol/L, sodium 136.9 mmol/L, potassium 3.25 mmol/L, chloride 104.7 mmol/L, blood urea nitrogen (BUN) 8.55 mmol/L, creatinine (Cr) 92.96 μmol/L, lactate dehydrogenase (LDH) 252 U/L, myoglobin 59.46 ng/mL, troponin I < 0.1 ng/mL, creatine kinase (CK) 132 U/L, and CK-MB 8 U/L. Coagulation function: PT 15.00 s, APTT 52.90 s, fibrinogen 2.41 g/L. Blood interleukin-6 (IL-6) was elevated (>5,000 ng/L). Full blood cell count, C-reactive protein (CRP), and procalcitonin (PCT) levels were monitored, with the results summarized in Table 1. The patient’s test for influenza B virus antigen, conducted outside of the hospital setting, yielded a positive result. Bedside cardiac ultrasound revealed no significant structural cardiac abnormalities. A lung CT scan showed multiple flaky, ground-glass opacities in both lungs (Figure 1). Upon admission, the patient was diagnosed with severe pneumonia, type II respiratory failure, acute respiratory distress syndrome (ARDS), septic shock, and acute kidney injury (AKI). Treatment with ceftriaxone 2 g QD and oseltamivir 150 mg twice daily was initiated. Given the current influenza season, it is not excluded to combine *Staphylococcus aureus* infection after influenza, and add linezolid 0.6 g Q12h for anti-infection treatment. Despite these interventions, the patient’s blood pressure dropped to a low of 80/40 mmHg within the first hour of admission. Norepinephrine and posterior pituitary hormone was given to maintain blood pressure. The dosage of norepinephrine was increased from 0.2/μg/kg/min to 2 μg/kg/min. Considering the presence of severe ARDS, a lung protective strategy was adopted by adjusting the ventilator parameters (tidal volume of 300 mL/kg, pressure support of 14 cmH_2_O, PEEP of 12 cmH_2_O, respiratory rate of 18 breaths/min, and FiO_2_ of 100%) to maintain a plateau pressure of ≤30 cmH_2_O, and pulmonary recuperation therapy was given. Four hours after admission, repeat blood gas analysis showed that pH was 7.12, PaO_2_ was 54 mmHg, PaCO_2_ was 60 mmHg, and the patient’s ARDS symptoms did not improve. The patient exhibited normal cardiac contractility and stable circulation. As a result, Venous–venous extracorporeal membrane oxygenation (VV-ECMO) therapy was initiated. A 17Fr arterial catheter was inserted 15 cm into the right internal jugular vein, and a 21Fr venous catheter was placed in the right femoral vein to the level of the inferior vena cava. The ECMO parameters were set to a rotational speed of 2,400 rpm, a flow rate of 3.2 L/min, FiO_2_ 100%, and an airflow rate of 3 L/min. The patient was admitted to the hospital with a presentation of anuria and metabolic acidosis. Consequently, Continuous renal replacement therapy was initiated to address the patient’s condition.

**Table 1:** The change of WBC, NE%, LY%, CRP, and PCT.

Laboratory variables	Frist day	Second day	Third day
PCT (ng/ml)	44.47	11.43	64.5
CRP (mg/L)	61.61	54.8	65.42
WBC (*10^9^/L)	1.18	0.5	1.01
NEUT (%)	23.8	32	57.2
LYMPH (%)	73.7	66	34.7

PCT, procalcitonin; CRP, c-reactive protein; WBC, white blood cell; NEUT, neutrophil; LYMPH, lymphocytes.

**Figure 2:**
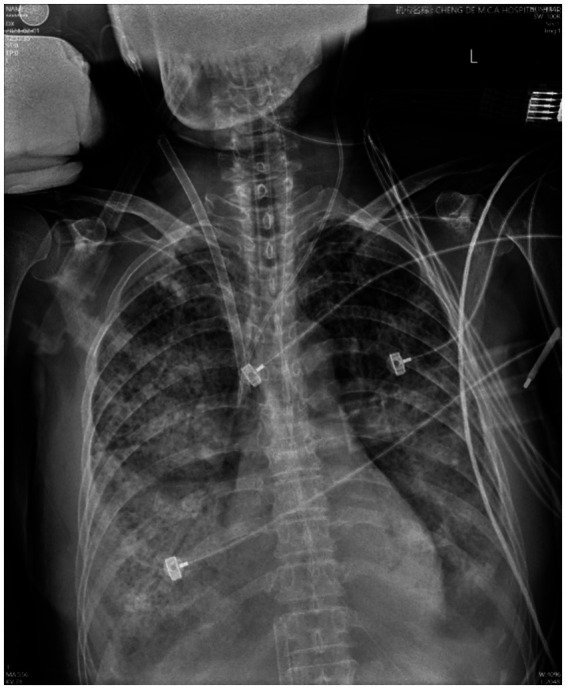
Chest X-ray images of the patient. The chest X-ray image, obtained on day 2, showed multiple patchy and flaky shadows in the lungs.

**Figure 3:**
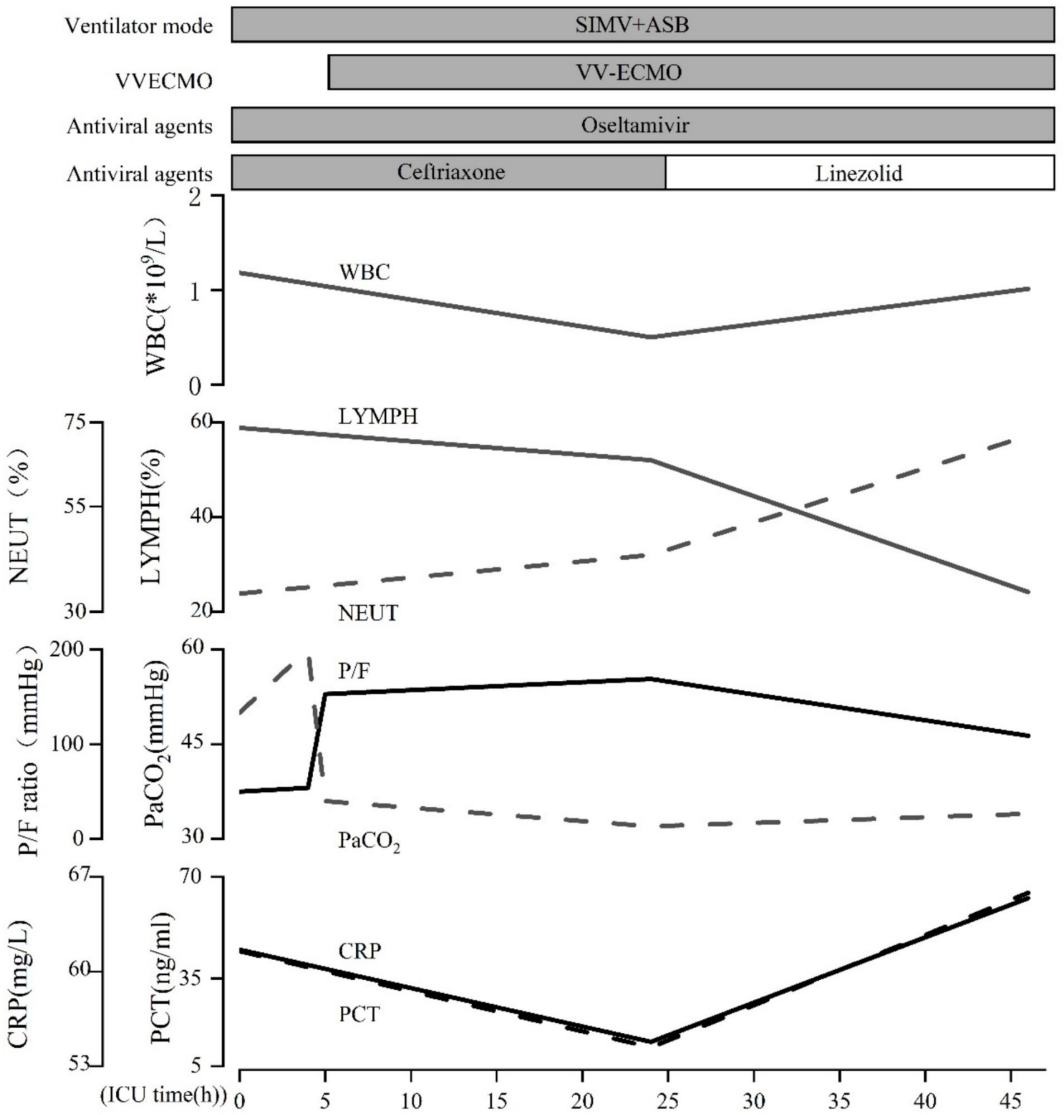
The patient’s clinical course.

On the second day of hospitalization, sputum and blood tested positive for influenza B virus nucleic acid and antigen. Sputum and blood culture results demonstrated gram-positive cocci. Based on the pathogenetic results, a diagnosis of severe pneumonia (influenza B virus infection) and septicemia (gram-positive cocci) was made, and the original dose of anti-infective regimen was continued. Despite ongoing treatment, the patient’s respiratory status remained critical, chest radiographs continued to show significant lung inflammation (Figure 2). Unfortunately, the patient’s cardiac ultrasound suggested diminished myocardial contractility with a cardiac output of 2.0 L/min and a stroke volume of 22 mL. The electrocardiogram (ECG) suggests the presence of Second-degree type I atrioventricular blockage. Biochemistry tests revealed lactate dehydrogenase (LDH) > 4,000 U/L, myoglobin 209.26 ng/mL, troponin I < 0.26 ng/mL, creatine kinase (CK) > 6,400 U/L, and CK-MB > 1,200 U/L. It was explained to the patient’s relatives that there is a possibility of myocarditis and septic myocardial injury and that the progression of the disease may necessitate treatment utilizing venous–arterial ECMO (VA-ECMO). Nevertheless, this course of treatment was declined, with conservative treatment instead being requested. Dobutamine was administered to enhance myocardial contractility. However, the patient’s cardiac contraction did not improve, and circulatory failure ensued. Finally, the patient succumbed to the combined effects of infectious shock and multi-organ failure 46 h after admission. Post-mortem sputum and blood cultures confirmed the presence of MSSA. Therefore, she was diagnosed with co-infection with the IBV and both pneumonia and septicemia caused by MSSA. The patient’s clinical course is shown in Figure 3.

## 3 Discussion

In recent years, *S. aureus* has emerged as a significant factor in co-infections with influenza viruses, contributing to the development of fatal pneumonia (3–5). Although several national and international reports have documented co-infection between IBV and *S. aureus*, there is limited information on the management of fatal pneumonia caused by MSSA infection following IBV infection. To the best of our knowledge, this case is the first documented instance of ECMO being used to treat severe pneumonia caused by the co-infection of IBV and MSSA in China. The patient experienced a sudden onset of necrotizing pneumonia that rapidly progressed. Two hours after admission, blood and sputum samples were collected, and the results revealed MSSA infection, accompanied by a positive nucleic acid test for IBV. This suggests that both the influenza virus and *S. aureus* contributed to the immunopathologic process underlying pneumonia.

There are three main mechanisms by which secondary *S. aureus* infections occur following influenza virus infection. First, the influenza virus triggers an excessive inflammatory response that damages the pulmonary epithelium, weakening local defense mechanisms and making the host more susceptible to bacterial pneumonia. The influenza virus can impair the bronchial and alveolar epithelium, exposing sites for bacterial adherence and reducing the respiratory tract’s ability to clear bacteria (6, 7). This facilitates the adherence and overgrowth of *S. aureus*. Second, IBV compromises the body’s innate immune function, preventing effective bacterial control. Neutrophils, which play a crucial role in bacterial clearance, are less effective during influenza infection. The influenza virus enhances susceptibility to bacterial infection by inhibiting the IL-17-mediated immune response, which impedes neutrophil recruitment (8, 9). Moreover, influenza infection can hinder the secretion of granulocyte colony-stimulating factor (G-CSF) and reduce myeloperoxidase (MPO) activity, resulting in dysfunctional neutrophil bacterial clearance (10). Additionally, influenza viruses impair the bactericidal capacity of neutrophils by inhibiting the activity of NADPH oxidase within these cells (11). Third, secondary *S. aureus* infections can also increase the virulence of the influenza virus. The bacterial proteases secreted by *S. aureus* can activate influenza hemagglutinin (HA), enhancing the pathogenicity of the virus (12).

The patient in this case was admitted with a rapidly deteriorating condition and unstable vital signs and was diagnosed with severe pneumonia and ARDS. Despite increasing the oxygen concentration, PEEP, and other ventilator parameters, respiratory failure did not improve. ECMO, however, offers a solution to the limitations of PEEP by facilitating effective blood gas exchange and tissue perfusion. ECMO is an adjunctive therapy that uses extracorporeal circulation technology to oxygenate blood via a membrane oxygenator and then pump it back into the body (13). Clinically, ECMO supports respiratory and/or cardiac insufficiency, providing time for functional recovery. ECMO can extend the survival of patients experiencing severe respiratory and circulatory failure (14). In this case, the patient had infectious shock and ARDS. ECMO helped enhance oxygen metabolism, maintain acid–base balance, and support carbon dioxide elimination, which contributed to the recovery of the cardiovascular system. According to established guidelines, ECMO is indicated if no contraindications are present and one of the following conditions is met: PaO_2_/FiO_2_ < 50 mmHg for more than 3 h, PaO_2_/FiO_2_ < 80 mmHg for more than 6 h, or arterial blood pH < 7.25 with PaCO_2_ > 60 mmHg for more than 6 h (15). After considering the patient’s condition, VV-ECMO was promptly initiated with the family’s consent. However, the patient’s condition continued to deteriorate rapidly, and she ultimately succumbed to multi-organ dysfunction. The challenges of treating this patient were considerable. The unknown etiology of the disease, its rapid progression, the presence of multiple organ failures, and the patient’s critical condition all contributed to the complexity of her management. If not addressed promptly, such factors could delay recovery and threaten the patient’s life. In this case, while the patient received active antimicrobial therapy, ECMO was initiated promptly to provide rest for the lungs, promote healing, and facilitate functional recovery. Despite these efforts, her condition continued to worsen, suggesting that in cases of severe pneumonia and septicemia caused by co-infection with the influenza virus and MSSA, a fatal outcome is likely.

This case still has some shortcomings. Due to the limitations of laboratory facilities, it was not possible to characterize the isolate molecularly and biologically to determine if it was positive for the PVL gene. Current research demonstrates that a correlation between *S. aureus* virulence and the expression of methicillin-resistant (i.e., MRSA) and toxin genes (e.g., PVL) (16). The presence of PVL-positive *S. aureus* has been associated with a poor prognosis, with a reported mortality rate of approximately 75% (17). In the future, it is expected that the detection of pathogenic virulence factors will be improved, thereby facilitating a more comprehensive understanding of MSSA and enhancing the prognosis of patients.

## 4 Conclusion

Given the synergistic pathogenic effects between IBV and concurrent bacterial infections, it is critical to raise awareness about *S. aureus* pneumonia that occurs following influenza. Early diagnosis, along with timely administration of antiviral treatments and prevention of secondary bacterial infections, can reduce morbidity and mortality. Furthermore, the early use of ECMO can help maintain respiratory and circulatory stability, delaying disease progression in ARDS caused by co-infection with IBV and MSSA. This approach provides more time for therapeutic interventions and recovery.

## Data Availability

The datasets presented in this study can be found in online repositories. The names of the repository/repositories and accession number(s) can be found in the article/supplementary material.
